# Patients’ Perceptions of Exercise-Based Cardiac Telerehabilitation after a Myocardial Infarction—A Qualitative Study

**DOI:** 10.3390/ijerph20075420

**Published:** 2023-04-06

**Authors:** Ulrika Nilsson, Birgitta Öberg, Maria Bäck

**Affiliations:** 1 Department of Occupational Therapy and Physical Therapy, Sahlgrenska University Hospital, 41345 Gothenburg, Sweden; 2 Department of Health, Medicine and Caring Sciences, Division of Prevention, Rehabilitation and Community Medicine, Unit of Physical Therapy, Linköping University, 58183 Linköping, Sweden; 3 Insitute of Medicine, Department of Molecular and Clinical Medicine, Sahlgrenska Academy, University of Gothenburg, 40530 Gothenburg, Sweden

**Keywords:** coronary artery disease, exercise-based cardiac rehabilitation, interviews, physical therapy, telerehabilitation

## Abstract

To be able to design telerehabilitation programs targeting the needs and preferences of end-users, patients’ in-depth perspectives are needed. To date, such studies are lacking and, therefore, the aim of the present study was to describe patients’ perceptions of performing exercise-based cardiac telerehabilitation after a myocardial infarction (MI). Individual semi-structured interviews were performed with 15 patients (3 women, median age 69 years) after an MI who had participated in exercise-based cardiac telerehabilitation for three months. The interviews were transcribed verbatim and analyzed with inductive content analysis. An overall theme was defined as “Cardiac telerehabilitation—a new alternative for exercising that is easily accessible and up to date”. Four categories, including “The important role of a physical therapist with expert knowledge”, “Prerequisites playing an important role in the willingness to participate”, “Making exercise accessible and adjustable” and “Inspiring future exercise”, and 15 subcategories were identified. Understanding the patient’s perspective is an important key to further improving and successfully implementing exercise-based cardiac telerehabilitation, as an alternative or adjunct to traditional, centre-based programs. The findings can serve to improve patient–physiotherapist interactions and to inform important aspects related to exercise, technology and a sense of security from an exercise-based cardiac telerehabilitation program.

## 1. Introduction

Cardiac rehabilitation (CR), following a myocardial infarction (MI), is an evidence-based comprehensive intervention aiming to achieve clinical stabilization, cardiovascular risk reduction, disability reduction, psychosocial and vocational support and lifestyle behavioural change, including patients’ adherence and self-management [1]. Centre-based exercise programs, supervised by physiotherapists, are consistently identified as a cornerstone of CR, often referred to as exercise-based CR (EBCR) [1]. The benefits of EBCR after an MI are well known, in terms of the reduced risk of cardiovascular mortality, hospital admission, the risk of recurrent MI and improved exercise capacity [2] and, as such, it has the highest class of recommendation and level of evidence in international guidelines [3,4].

Despite strong evidence-based benefits, centre-based EBCR remains underused in patients after an MI in Sweden and elsewhere [5,6]. Barriers related to non-attendance are already known; they include long distances, transportation to the hospital and work-related factors [7,8]. Unsupervised home-based EBCR programs in the early stage after an MI may increase access but can be criticized for lack of supervision during exercise and safety issues, as well as difficulties in optimally prescribing and progressing exercise programs [1]. Digital health interventions, such as telerehabilitation, provide an opportunity to redesign and expand evidence-based care in new settings by combining the accessibility of home-based EBCR with specialist monitoring, interaction and support of centre-based EBCR [9,10]. Studies using cardiac telerehabilitation to improve outcomes related to physical fitness and physical activity are promising, but yet small and lack data on recurrent events, rehospitalization, mortality and sufficient safety reporting [9,11,12]. In addition, the telerehabilitation models used so far are heterogeneous, typically delivered as heart rate or ECG-monitoring with data uploaded to web applications and with feedback provided through phone, e-mail or short message service once a week by healthcare providers [12]. As such, studies using cardiac telerehabilitation models with real-time exercise sessions and direct feedback and interaction with patients still need to be explored.

To facilitate the further development and implementation of exercise-based cardiac telerehabilitation programs into real-world practice, increasing knowledge of patients’ perceptions is important [13]. To date, qualitative studies have frequently been excluded from previous systematic reviews in this field and they are the least used approach to evaluate cardiac telerehabilitation programs [9,11,13]. A recently published scoping review used the technology acceptance model to map available research on patients’ technology acceptance of comprehensive home-based cardiac telerehabilitation in patients with coronary artery disease (CAD) [13]. In this review, only three of the included studies used qualitative methods and they did not specifically focus on exercise [13]. Consequently, there is a need to further explore patients’ in-depth perceptions of exercise-based cardiac telerehabilitation, which is key to being able to design and deliver programs targeting the needs and preferences of patients. The aim of this study was, therefore, to describe patients’ perceptions of performing exercise-based cardiac telerehabilitation after an MI.

## 2. Materials and Methods

### 2.1. Design

This is a qualitative study with individual semi-structured interviews, analysed using inductive content analysis [14].

### 2.2. Patients

Inclusion criteria: a diagnosis of MI and age ≤ 80 years at Sahlgrenska University Hospital (SU) Sahlgrenska and Östra, Southern Älvsborg Hospital Borås (SÄS) and Skaraborg Hospital Skövde (SKAS). Exclusion criteria: incomplete revascularization, significant valve disease, severe arrhythmia, heart failure (New York Heart Association classification, NYHA, 3b-4), difficulty speaking the Swedish language, no internet connection or access to a device.

### 2.3. Setting

This study was part of a larger research program, also involving a quantitative evaluation of exercise-based cardiac telerehabilitation delivered in real-time, group-based videoconferencing meetings under the direct supervision of a physiotherapist. The physiotherapist used a PC with videoconferencing equipment and patients used their own devices, such as a PC or tablet. Before entering EBCR, patients performed a pre-exercise assessment, including tests of physical capacity, in accordance with routine EBCR in Sweden [15]. All the patients had undergone an adequate risk assessment and a cardiologist was responsible for medical safety. The exercise-based telerehabilitation program consisted of 60 min of aerobic and strength exercises twice a week for three months and it was individually adapted to each patient.

After a warm-up period, the aerobic exercises were performed in 45 sec intervals with a high intensity level corresponding to 14–17 on a Borg rating of perceived exertion scale (RPE) with 15 s periods at moderate intensityRPE 12–13) between intervals for a total duration of 25 min. The muscular resistance exercises were performed in 12 reps × two sets and consisted of four exercises with an elastic band and six exercises for the core and arms performed on a mat. The exercise program ended with a cool-down period and exercises for flexibility.

Before and after each session, patients were given the opportunity to talk individually to the physiotherapist. The interviews were performed when the patient had finished the program.

### 2.4. Procedure

Eligible patients were informed about the study by a physiotherapist working at the cardiac intensive care unit or on the first CR visit. Patients who agreed to participate gave their written consent prior to the start of the study. Of 23 patients who had completed the cardiac telerehabilitation program, 15 patients were purposively selected to participate in the interviews. The purposive selection was made to obtain as much variation, including gender, age and different hospitals, as possible. The sample size in a qualitative study depends on the quality and richness of the data and it is not possible to propose a specific number of interviews [16,17]. In this study, after having performed 15 interviews, no new information emerged.

The interviews were performed by the last author (MB), who has previous experience in both interviewing and EBCR. The interviews were conducted digitally or in a private room at the hospital, as preferred by the patient. All the interviews started with the question “Can you tell me about your perceptions of participating in exercise-based cardiac telerehabilitation?”. The interviews had a semi-structured focus and were based on an interview guide (Appendix A). Follow-up questions were used to deepen the dialogue. One pilot interview was performed to test the interview guide. No changes were needed and the pilot interview could be included in the analysis. The interviews were performed between 26 June 2019 and 28 August 2020, lasted for a median of 38 (min 20 and max 56) minutes and were recorded with a digital voice recorder. The interviews were transcribed verbatim.

The study was conducted according to the guidelines of the Declaration of Helsinki and approved by the Regional Ethical Review Board in Gothenburg (registration no: 790-18).

### 2.5. Data Analysis

The results were analysed using qualitative content analysis according to Graneheim and Lundman [18]. Qualitative content analysis provides a systematic and objective way to make valid inferences from written data. The purpose is to deliver understanding and knowledge of the event under study. The qualitative content analysis includes both latent and manifest content, where manifest analysis describes the obvious components, while latent analysis means the interpretation of the underlying meaning of the text [18].

The analysis followed The COnsolidated criteria for REporting Qualitative research (COREQ) guidelines [19]. First, the transcribed interviews were listened to and read several times to attain a sense of the whole. The text was then divided into meaning units, which compress related text content connected to the aim. The meaning units were condensed and labelled with a code. The codes were then sorted into subcategories and categories depending on their similarities and differences. An example of the data analysis process is found in Table 1.

A first analysis, from reading the interviews in their entirety and dividing the text into meaning units, condensed meaning units and creation of codes, was performed by the first author (UN). A second analysis, including the same steps, was performed in the first five interviews by MB and the results were compared. UN and MB then worked together to define preliminary subcategories and categories, which were then discussed in an iterative process with a third physiotherapist (BÖ) until a consensus was reached. Both UN and MB are physiotherapists and have a pre-understanding of the subject through clinical experience with the patient group and by performing the exercise-based cardiac telerehabilitation program during the study. BÖ has no pre-understanding of the concept of telerehabilitation or the patient group but had experience with the method of qualitative content analysis.

## 3. Results

Among the 15 included patients (3 women and 12 men), 1 patient was from SKAS, 8 patients from SU Sahlgrenska, 4 patients from SU Östra and 2 patients from SÄS. Their median age was 69 (min 51 and max 79) years.

The overall theme that emerged from the analysis was: “Cardiac telerehabilitation—a new alternative for exercising that is easily accessible and up to date”. In general, patients perceived that exercise-based cardiac telerehabilitation, with real-time exercise sessions supervised by a physiotherapist, was a modern form of exercising that felt safe and was easy to perform in a time-efficient way.

An overview of the 4 main categories and 15 subcategories and the overall theme is presented in Table 2.

### 3.1. The Important Role of a Physiotherapist with Expert Knowledge

Patients stated that the physiotherapist played an important role in their participation in exercise-based cardiac telerehabilitation. They said that the workout felt safe, that they received clear instructions and had the opportunity to ask questions and receive advice. Moreover, the patients perceived that the physiotherapist increased their motivation to exercise with a positive and encouraging attitude.

#### 3.1.1. Provides Safety

Patients stated that exercising in real-time with a physiotherapist during the exercise-based cardiac telerehabilitation sessions gave them a sense of security in case an adverse event were to occur. Another aspect of security was that the physiotherapist knew their address in case of a serious adverse event during the exercise session. Patients felt that it was important that the physiotherapist had disease-specific expertise and knew how to prescribe safe exercises.

“*It’s a sense of security, that someone who actually has in-depth expertise and knows what I need to exercise in terms of fitness and strength, both how much and what is best for me, because I’m not fully aware of that*.” (7)

The patients also thought about their own safety while performing home-based exercises after the MI.

“*I think more about safety, during exercise, I unlocked the door at home, because I did not know how my body would react*.” (8)

#### 3.1.2. Encouraging

Patients perceived that the physiotherapist was professional, encouraging, positive and motivating during the exercise-based cardiac telerehabilitation sessions, which gave them the extra push they needed, to maintain a higher intensity and not give up.

“*I think it’s necessary to have a physiotherapist who motivates you by your side for a long time. It’s a bit of encouragement, I think it’s very important*.” (13)

#### 3.1.3. Need for Clear Instructions

One important aspect while exercising via telerehabilitation was that the instructions from the physiotherapist were clear and uncomplicated and easy to follow.

“*Yes, she [the physiotherapist] was very clear, she knows what she is doing and talked and explained why we did what we did, so that was great*.” (5)

Some patients said that it was not a problem that different physiotherapists conducted the exercise sessions, while others felt that it was a bit more difficult to follow the new physiotherapist digitally if the exercises were not performed in exactly the same way.

“*We have two different physiotherapists and they have slightly different styles and it is also good that they have different balance exercises, it’s good to have different versions*.” (1)

“*If there is a new instructor who performs another exercise or does it another way, then I have to check again and then I can’t keep up*.” (3)

#### 3.1.4. The Importance of Interaction

The opportunity to ask questions and receive support from the physiotherapist was regarded as an important part of the exercise-based cardiac telerehabilitation program. Some patients said that it was not always easy to ask questions digitally, as compared to a real-life session.

“*Yes, you can ask questions afterwards, if someone asks a more personal question, then the physiotherapist says, ‘Yes, we can talk about that afterwards’ and it also feels very good to be able to do that*.” (1)

Receiving feedback and advice from the physiotherapist about exercise was also described as a valuable aspect. Patients felt that it was not always easy to exercise on their own without support from the physiotherapist, as this often resulted in a more ineffective workout.

“*When I have to exercise myself, it becomes so much more inefficient. The breaks between the intervals are far too long and, although I can see the exercise instructions and read the text, it becomes stagnant*.” (6)

### 3.2. Prerequisites Playing an Important Role in the Willingness to Participate

Previous exercise habits and computer experience played a significant role in the patient’s willingness to participate in exercise-based cardiac telerehabilitation.

#### 3.2.1. Perceptions and Expectations Are Important When It Comes to Participating in Telerehabilitation

Patients said that they generally had a positive perception of exercising. Their expectations of the exercise-based cardiac telerehabilitation program were high, but they were usually met.

“*I thought it worked out very positively, smoothly and easily. I have always been a little fascinated by new thinking and new stuff, so, when I got the question about the study, I said yes and I had a positive input to this, so to speak, but I also think it has met the expectations I had*.” (7)

Obtaining information about exercise-based cardiac telerehabilitation at the first in-person meeting with the physiotherapist was an important and motivating aspect for patients who felt hesitation about participating.

“*I am an old man when it comes to electronics and computers and everything like that. I don’t really agree with all this modern technology, there was resistance, but the physiotherapist managed to convince me to try*.” (6)

#### 3.2.2. Fear of Movement

A perceived fear of movement in relation to exercising could be expressed by patients after their MI. Patients were more observant of bodily symptoms and they feared a recurrent MI, especially during exercises at a higher intensity.

“*Every time I make an extra effort, there is always a slight worry that has not existed before*.” (3)

Others felt no uncertainty or distress in relation to exercising and had no fears or perceived any obstacles to performing exercise-based cardiac telerehabilitation.

“*I was never afraid of having another heart attack, rather the opposite, it may have been a push in the back that you can now actually start a little aerobic exercise*.” (9)

#### 3.2.3. The Impact of Previous Experience of Exercise

Patients with previous experience of exercise were able to recognize several exercises in the telerehabilitation program, which was perceived as a facilitating factor.

“*I have performed many of these exercises previously, so I recognized a lot of it. So, for me, there was nothing strange*.” (14)

#### 3.2.4. Significance of Computer Skills

Some patients said that they used computers at work or during their leisure time. Those who did not have sufficient computer skills turned to relatives for help. Patients said that they understood that a lack of computer knowledge might be an obstacle to participating in exercise-based cardiac telerehabilitation.

“*I use a computer a lot at work, so the technical part wasn’t an obstacle for me, but I can imagine that many people will think it is*.” (1)

### 3.3. Making Exercise Accessible and Adjustable

Patients said that it was essential that exercise-based cardiac telerehabilitation was easily accessible to save time. Individualized exercise and user-friendly technology were also factors of relevance to patients.

#### 3.3.1. Availability

Patients said that the availability of home-based exercise was a great advantage, without the need for transport to the hospital and finding a parking space.

“*It easily takes two, two and a half hours if you don’t do the exercise at home and therefore I think this is great, because it cuts the timeframe*.” (7)

The fact that the exercises could be carried out in a small area without any equipment, except for elastic bands, was valued by the patients.

“*The advantage of this is that you don’t need any equipment, more than an elastic band, so, wherever you go, you can just bring an elastic band and your pants and a sweater*.” (4)

Some patients valued a scheduled time for the exercise sessions, while others could perceive negative aspects about having a predetermined time, as it was difficult to be absent from work.

“*If you have already booked exercise at a certain time, it’s much easier to just do it, than if I have to think, when should I exercise*?” (3)

#### 3.3.2. Feeling of Being a Group Member or a Single Participant

Some patients stated that, even though they performed home-based exercise, they felt like the exercise program was carried out together as a group. Exercising with peers was important to play down the dramatic feeling of exercising after an MI.

“*You feel like you’re a group, even though I don’t know exactly what everyone looks like, you know that there are others out there doing the same thing as me. That’s quite fun, I think*.” (9)

Other patients felt that the exercise sessions were more like a private session between themselves and the physiotherapist. They wanted to focus on their own performance and did not bother about the other patients.

“*I did not experience it as a group really, it was like between me and the person who led it, so to speak. I knew there were others participating, but I didn’t really care about that, except that I sometimes saw them*.” (14)

#### 3.3.3. The Opportunity to Self-Regulate Exercise

Patients perceived that the exercises felt personalized, based on their own ability and previous experiences, despite being performed in a group.

“*Yes, it went well, I think. Since these are the exercises that everyone does the same amount of and she [the physiotherapist] says that, if you can’t go all the way, then adapt like this and so on*.” (4)

#### 3.3.4. User Acceptance

The technology was generally seen as user friendly. Some patients said that user acceptance was possibly a generational issue.

”*For my generation and those who are older, I think you perhaps need to be a little more hands-on and show how it works on a computer*.” (1)

Patients could experience some initial frustration with video and sound settings and not understanding the digital terminology, but they quickly learned how to manoeuvre the technology and they then found it user friendly.

“*It has worked very well, it was just a question of getting started and then, when you figured out how to use the settings, it was very simple, no problems. I got the link and just clicked on it and got started*.” (5)

Some patients wished that they had received more information and instructions about the technology before starting the exercise program, to learn about settings and avoid technical problems.

“*The first time, you should perhaps allocate a small section of 15 min where you show how it works, this is how you ‘pin’ and here is the sound*.” (7)

#### 3.3.5. Simple, Enjoyable Exercises Contributed to Implementation

Patients stated that they perceived exercise-based cardiac telerehabilitation as a new, well-conducted concept and a fun and different way of exercising. It was also simple and easy to carry out.

“*It has been very good, I think, and in a form that suits me very well*.” (5)

Even though the limited space of home-based exercise was seen as a potential limitation for implementation, the overall patient perception was that the program had a wide variety of exercises that included the whole body.

“*The exercises you can do are a bit limited, of course, so you must be a little clever. But I think it has worked well. I can definitely imagine doing more exercises via telerehabilitation*.” (3)

### 3.4. Inspiring Future Exercise

Patients said that they observed positive effects of the exercise-based cardiac telerehabilitation program which motivated them to continue exercising.

#### 3.4.1. Perceived Benefits of Exercise

The patients described a positive feeling and increased strength and fitness fairly quickly. They noticed that they were able to perform the exercises more and more effectively as the exercise period progressed. Even their family members were able to see the positive effects of exercising.

“*So, all of a sudden, I discovered that I like exercising, so I now find that I am just getting better and better and both my wife and my children can see that I have improved*.” (15)

#### 3.4.2. Inspiration for Future Exercise

After having finished the exercise-based cardiac telerehabilitation program, patients expressed the feeling of being motivated and having enough knowledge to be able to continue with regular exercise, even though some patients would have liked to continue with cardiac rehabilitation.

“*Yes, I would like to continue, but my exercise period is over, I understand. But then it is good that I have already established a routine to continue on my own*.” (13)

Others felt that continued exercise could be a challenge and they wanted further advice from the physiotherapist.

“*Some tips on what exercises I can do on my own, when you can’t go to the gym, I need that. As well as what kind of aerobic exercise I can do myself, either at home or outside or wherever*.” (9)

## 4. Discussion

This study contributes to an increased understanding of patients’ perceptions of performing exercise-based cardiac telerehabilitation after an MI. The overarching theme indicated that exercise-based cardiac telerehabilitation is a modern form of exercising that is easily accessible, time efficient, up to date and easy to perform.

The information about exercise-based cardiac telerehabilitation provided by physiotherapists at the CR centre was described as important for participation, especially by patients who were insecure about digital healthcare. This finding is in line with a study by Frederix et al. [10] showing that education by a knowledgeable professional could be a prerequisite for patients to participate in digital health programs. As worries about perceiving a poorer level of care by using digital services have been reported, it is important in the future to inform patients of the potential benefits of telerehabilitation in order to overcome any barrier or major challenges when it comes to incorporating digital health in daily clinical practice [10,20].

According to guidelines, EBCR programs should be individually prescribed, based on the initial evaluation of physical capacity to achieve the optimal effect [1]. One important finding in the present study was that patients perceived the exercise-based cardiac telerehabilitation program as individualized and that they were able to adapt the exercises according to their own physical capacity. The importance of individualized exercise prescription is further supported by Maddison et al. [21], showing that patients with CAD performing a telerehabilitation program via an established CR clinic reported a similar increase in VO_2_max as centre-based EBCR [21].

In line with previous studies [13,22], patients in the present study described the interaction with the supervising physiotherapist as a central factor when it came to being able to maintain the prescribed intensity and duration of the exercises. This is an important clinical message as Xia et al. [23] have shown that home-based CR is usually limited to written instructions, without sufficient feedback from physiotherapists, which leads to the questioning of the effectiveness of such programs. Moreover, a recent qualitative study of attitudes to physical activity after MI found that a lack of guidance could result in a loss of confidence and a feeling of vulnerability regarding exercising [24]. Fear of movement (kinesiophobia) is commonly reported by patients who have suffered an MI [25,26]. A previous study has found that non-consistent, unclear information about physical activity and exercise increased fear of movement [27]. Similarly, some patients in the present study expressed fear of movement in relation to exercising after an MI, but the overall perception was that the fear of movement was not a barrier to performing the exercise-based cardiac telerehabilitation program, as patients felt secure due to the supervising physiotherapist.

In a previous study, patients stated that social support, in terms of peers sharing experiences, was a significant prerequisite for attending centre-based EBCR programs [22]. The importance of feeling supported by the direct interaction with healthcare providers and peers, resulting in subsequent psychological and motivational benefits, has also been highlighted in previous studies [11,12]. One interesting finding in the present study was that patients found it possible to choose whether they wanted to feel part of group-based exercise or whether they preferred to see themselves as single participants without any focus on others. In this way, exercise-based cardiac telerehabilitation can be a person-centred and appealing model for most patients.

Patients in the present study stated that the accessibility of telerehabilitation was a motivating factor, which is supported by previous studies [11,12]. Scheduled exercise sessions made sure that the exercise was performed, but this could also conflict with working commitments. This factor is in line with a previous study [22] and, for the future successful implementation of exercise-based cardiac telerehabilitation, several exercise groups at different times during the day could be recommended.

In the end, the successful incorporation of exercise-based cardiac telerehabilitation into clinical practice is dependent on patients accepting the intervention. External variables such as ease of system use, learnability and technology self-efficacy may influence acceptability [13]. In general, the patients in the present study perceived sufficient acceptability and usability with the exercise-based cardiac telerehabilitation program but described a learning process, in terms of both managing the digital platform and performing the digital exercise program.

### Methodological Considerations

In terms of credibility, participants need to understand the event under study and the fact that it represents a diversity of demographics, viewpoints and experiences [17,18,28]. Even though we used purposive sampling, the included patients may represent a homogeneous group. Neither occupational status, nor earlier exercise habits or computer skills could be included in the strategic selection, due to a lack of information. In addition, as a greater proportion of the included patients were men, this must be considered in terms of the transferability of the results. Even though the present context needs to be taken into consideration when interpreting the results, we believe our findings are relevant to similar settings.

Another aspect of credibility is that data analysis was planned a priori [18]. Investigator triangulation with an external assessor was used during the analysis, seeking agreement between all the co-authors [28,29]. Direct quotes from the interviews are used to exemplify the interpretations found by the authors [18,28]. We have considered the risks of social desirability, as the interviewer only performed a small number of exercise sessions and did not interact with the patients on more than a few occasions.

In summary, to meet the needs of more patients, new evidence-based best practice models for EBCR must be evaluated and implemented. To succeed, it is important to keep patients at the centre of the development of digital care models, to achieve a patient-centred healthcare system and to guarantee results that are practical, interpretable and comprehensive. The present study adds several aspects that are important to consider when setting up an exercise-based cardiac telerehabilitation program. The interaction with an expert physiotherapist played an important role for patients, and thus models without real-time feedback may be less successful. Patients appreciated the possibility of being able to choose settings in the telerehabilitation platform to either obtain a strong sense of group dynamics and interaction with peers or to focus on their own exercise. Regardless of this choice, it is important for physiotherapists to give clear instructions and adapt the exercise program to individual patients. Based on the fact that patients also felt safe and motivated during exercise sessions and managed to handle the technology after an initial learning period, telerehabilitation can be considered a promising model.

However, although a growing amount of research is being conducted on the evaluation of new digital delivery models, it is important that this rapid digital evaluation should not replace traditional care but rather supplement it. Hybrid models of EBCR, including components from both centre- and home-based EBCR, are being increasingly suggested [30]. In the future, the ultimate choice of EBCR delivery is likely to depend on the individual patient’s preference.

## 5. Conclusions

This study showed that patients perceived exercise-based telerehabilitation as a modern and easily accessible way of exercising after an MI. Patients valued the supervision and support from physiotherapists with expert knowledge in EBCR which provided a sense of security during exercising. Understanding the patient’s perspective is an important key to further improving and successfully implementing exercise-based cardiac telerehabilitation, as an alternative or adjunct to traditional, centre-based EBCR. The findings may serve to improve patient–physiotherapist interactions, as well as inform important aspects related to exercise, technology and a sense of security to inform the design, development and evaluation of future exercise-based cardiac telerehabilitation programs, with the overall aim of increasing patient-centred care and accessibility.

## Figures and Tables

**Table 1:** Example of the data analysis process.

Meaning Unit	Condensed Meaning Unit	Code	Sub-Category	Category
Can you tell us about your perceptions of exercising via telerehabilitation? I think it has been very positive. I see it as a very big advantage that you can do it at home. I live in the country and have about 15 km to the nearest gym, so this is a very convenient way.	Positive, I see it as an advantage to exercise at home.	Exercising at home	Availability	Making exercise accessible and adjustable

**Table 2:** Overview of categories, subcategories and overall theme.

Theme	**Cardiac telerehabilitation—a new alternative for exercising that is easily accessible and up to date.**			
Category	The important role of a physiotherapist with expert knowledge	Prerequisites play an important role in the willingness to participate	Making exercise accessible and adjustable	Inspiring future exercise
Sub-category	Provides safety Encouraging Need for clear instructions The importance of interaction	Perceptions and expectations are important when it comes to participating in telerehabilitation Fear of movement The impact of previous experience with exercise Significance of computer skills	Availability Feeling of being a group member or a single participant The opportunity to self-regulate exercise User acceptance Simple and enjoyable exercises contributed to implementation	Perceived benefits of exercise Inspiration for future exercise

## Data Availability

The interviews analysed in this study are not publicly available due to identifying patient data that should not be shared. Upon reasonable request, de-identified data may be available from the corresponding author.

## Appendix A

Interview guide

- Can you tell me about your perceptions of participating in exercise-based cardiac telerehabilitation?
- Which factors have been important for you when performing the exercise-based telerehabilitation program?
- Did you perceive anything that was particularly positive in connection with the exercise-based telerehabilitation?
- Did you perceive any obstacles or concerns in connection with exercise-based telerehabilitation?
- How did you experience exercise-based telerehabilitation compared with previous exercise experiences?
- How did you perceive the interaction with the physiotherapist during the exercise-based telerehabilitation sessions?
- How did you perceive the interaction with peers during the exercise-based telerehabilitation sessions?
- How do you perceive the physiotherapist’s role in exercise-based telerehabilitation after a myocardial infarction?
- Now that you have completed the exercise program, do you need any support to be able to continue exercising?
